# Human-mediated admixture shapes high genetic diversity and the invasion dynamics of *Lupinus nootkatensis* in Iceland

**DOI:** 10.1038/s41598-026-54580-3

**Published:** 2026-05-22

**Authors:** Magdalena Szenejko, Przemysław Śmietana, Remigiusz Panicz, Jakub Skorupski

**Affiliations:** 1 Institute of Marine and Environmental Sciences, University of Szczecin, Mickiewicza 16 St., 70-383 Szczecin, Poland; 2 The Centre for Molecular Biology and Biotechnology, University of Szczecin, Wąska 13 St., 71-415 Szczecin, Poland; 3 Faculty of Food Sciences and Fisheries, West Pomeranian University of Technology in Szczecin, Kazimierza Królewicza 4 St., 71-550 Szczecin, Poland

**Keywords:** Cryptic diversity, Human-mediated admixture, ISSR, *Lupinus nootkatensis*, Propagule pressure, Spatial genetic structure, Ecology, Ecology, Evolution, Genetics, Zoology

## Abstract

The invasion of the Nootka lupin (*Lupinus nootkatensis*) in Iceland presents a fascinating scenario: an introduced species on an isolated island that retains exceptionally high genetic diversity. Using inter-simple sequence repeat (ISSR) markers, we analysed the population structure of this widespread coloniser to understand the mechanisms behind its success. Contrary to expectations of a founder effect, our results reveal substantial polymorphism (100% at the dataset level) and high gene diversity, indicating that high propagule pressure from repeated, large-scale introductions has effectively countered genetic bottlenecks. Spatial analyses (PCoA, AMOVA) demonstrated a highly admixed structure where genetic clustering explains twice as much variance as geographical regions (12.3% vs. 5.9%). Importantly, a Mantel test confirmed a remarkably weak isolation-by-distance signal (explaining around 6% of the variance), aligning directly with the AMOVA results. This structural concordance shows that human-mediated transport—particularly from Reykjavík, the demographic hub—has completely overridden natural spatial gradients. We conclude that historical land reclamation efforts have acted as massive hyper-vectors, mixing diverse lineages to form a highly resilient, interconnected metapopulation. Therefore, effective management should shift from broad-scale eradication to targeted biosecurity measures that limit further human-facilitated mixing of these distinct genetic lineages.

**Supplementary Information:**

The online version contains supplementary material available at 10.1038/s41598-026-54580-3.

## Introduction

Nootka lupine, *Lupinus nootkatensis* Donn ex Sims, 1810 (Fabaceae), has become the dominant invasive species in the Icelandic sub-arctic ecosystem^1^. Its noticeable dominance and ability to transform landscapes at a large scale make it a key subject for studying the ecological effects of human-introduced plants^2^. The species acts as a significant ‘ecosystem engineer’ ^3^; by fixing atmospheric nitrogen and altering soil chemistry, it permanently transforms native moss-heathlands into dense, monospecific thickets. Moreover, a single plant can produce over 2,000 seeds in one season, spreading to new areas through water, landslides, storms and over longer distances by birds^4–6^. As a result, the Nootka lupine exemplifies a conflict species, highlighting the complex trade-offs between historic ecosystem restoration and contemporary biodiversity conservation^3,7–9^.

*L. nootkatensis* is a perennial legume native to north-western North America. Its widespread presence in Iceland results from a complex history of intentional, recurring, state-led introductions. The species was first brought to the island in 1885 as an ornamental plant^10^, during a period of extensive trials testing exotic species for Icelandic horticulture^6,11,12^. During these initial trials, *L. nootkatensis* was sown alongside several other lupine species. These early seeds likely originated from breeders in Norway or Sweden, with England considered the probable primary source of the species in Scandinavia^5,13,14^.

A second, much larger-scale initiative was launched in 1945 specifically for land reclamation and soil stabilisation, led by the Icelandic Forest Service (IFS) and the Soil Conservation Service of Iceland (SCSI)^15^. Importantly, the scale of these efforts increased substantially over the following decades. Around 1975, methods for using lupine to reclaim eroded lands were systematically developed^16^. By 1986, the SCSI had established dedicated lupine seed-harvesting fields (e.g., in Heiðmörk), leading to extensive, island-wide sowing campaigns in the 1990s^12^. Although most intensive sowing took place before 2000^17^, official reclamation activities using this species continued until 2018^18^. This deliberate, continuous, and large-scale introduction campaign effectively bypassed typical genetic bottlenecks, providing the species with a broad genetic base through exceptionally high propagule pressure from diverse sources. Despite extensive ecological and management-focused research^2,7,19–24^, the population genetic structure of *L. nootkatensis* in Iceland remains poorly resolved.

Genetic assessments reported so far showed relatively low differentiation among Icelandic populations. For instance, studies using SNP (Single Nucleotide Polymorphisms) markers derived from the evolutionarily conserved nuclear ITS2 (Internal Transcribed Spacer) region confirmed species identity but lacked the resolution to detect recent intraspecific divergence^25^. Similar limitations were observed in studies of *L. polyphyllus* on the East European Plain, where ITS1–2 markers remained uninformative, whereas hyper-variable markers revealed significant variation^26^. These findings highlight the need to use high-resolution molecular tools to accurately evaluate the evolutionary potential and spatial dynamics of such recent biological invasions.

Inter-Simple Sequence Repeat (ISSR) markers offer a practical and high-resolution method for reassessing genetic diversity in non-model invasive plants. ISSR markers target highly variable inter-microsatellite regions, requiring no prior genomic information and producing high-resolution banding patterns that often exceed 90% polymorphism^27–29^. This makes them particularly effective for exploratory screening and detecting fine-scale admixture in species where native-range reference data are limited^30,31^.

Therefore, this study aims to thoroughly reassess the invasive potential and genetic structure of *L. nootkatensis* across Iceland. By expanding the analysis to 33 populations representing the species’ main range, we address the following specific objectives: (1) to measure the current level of genetic diversity and ascertain whether the invasion is genetically depleted or diverse; (2) to determine if the population structure follows a natural geographical gradient or a complex mosaic pattern indicative of human-mediated dispersal; and (3) to establish a reliable genetic baseline for future monitoring and management strategies.

## Results

### ISSR amplification profiles and marker informativeness

Eight ISSR primers generated 257 clear and scorable loci across the 33 *L. nootkatensis* sampling sites (hereafter ‘populations’, each represented by a pooled DNA sample from five plants) (Table S1). All loci were polymorphic at the dataset level (100% polymorphism). Fragment sizes ranged from ca. 200 bp (primer 2) to 2816 bp (primer 807) (Table 1). Primer 807 yielded the highest number of loci (42) and the highest number of population-specific bands (14), whereas primer 817 produced the fewest loci (26) and nine population-specific bands. Across primers, the mean number of loci per primer was 32.1, and the mean PIC value was 0.188, ranging from 0.152 (primer 807) to 0.213 (primer 7). Representative ISSR profiles obtained with primer 807 are shown in Fig. S1. Across all primers, 74 loci were population-specific, meaning they were detected in only one population and absent from the remaining populations (Table 1). Population-specific bands occurred across all primers, with primer 807 contributing the largest share. One population on the Southern Peninsula, population 9 (Grindavík), contributed 10 population-specific bands, representing 13.5% of all population-specific loci detected across Iceland. These population-specific loci were amplified by primers 5, 7 and 810. For Grindavík, primers 7 and 810 produced unique fragments of ca. 1156 bp and ca. 1042 bp, respectively.

**Table 1:** Amplified products obtained with ISSR primers for *L. nootkatensis* populations.

Primer name	Size range (bp)	Total number of products	PB	PPB (%)	UB	PIC
2	200–1947	29	29	100	10	0.156
5	287–2034	39	39	100	9	0.199
7	265–1829	27	27	100	7	0.213
M09	284–1706	32	32	100	6	0.204
807	293–2816	42	42	100	14	0.152
810	285–2218	30	30	100	10	0.186
817	279–1956	26	26	100	9	0.192
888	278–1956	32	32	100	9	0.205
Total (mean per primer)	200–2816 (271–2058)	257 (32.1)	257 (32.1)	**-** (100.0)	74 (9.3)	**-** (0.188)

PB number of polymorphic bands, PPB the percentage of polymorphic bands, UB number of unique bands, PIC value of Polymorphism Information Content

### Genetic diversity across populations

All loci scored in each population were polymorphic, with an average of about 36 per population (Table 2). Band richness in pooled population profiles, expressed as the number of loci detected per population profile (P), varied widely from 17 to 74. Population 9 (Grindavík) showed the highest number of detected loci (74), followed by populations 27 (51), 26 (47) and 4 (44). In contrast, populations 17 and 18 in the Western Region showed markedly fewer loci (17 and 28, respectively). The assay efficiency index (AEI) was 32.1 and the percentage of polymorphic loci across the dataset (%P) was 100.0. The mean polymorphism information content (PIC) across loci was 0.188, with locus-level values ranging from 0.058 to 0.500. Shannon’s information index (I) averaged 0.270, ranging from 0.180 in population 17 to 0.358 in population 9. Pairwise Dice similarity values ranged from 0.098 to 0.606 (mean 0.306), indicating moderate overall similarity combined with pronounced heterogeneity among populations (Tables 2 and S2). The highest similarity was observed between populations 5 and 6 (Si = 0.606). Other high-similarity pairs included populations 12 and 13 (Si = 0.600), 30 and 32 (Si = 0.567), 13 and 18 (Si = 0.565), 26 and 27 (Si = 0.551), 5 and 7 (Si = 0.531) and 8 and 10 (Si = 0.531). The lowest similarities included population pairs 9 and 18 (Si = 0.098), 21 and 25 (Si = 0.102), 3 and 25 (Si = 0.125), 18 and 30 (Si = 0.125), 3 and 18 (Si = 0.127), 1 and 18 (Si = 0.127) and 1 and 33 (Si = 0.133). Several populations appeared repeatedly among the lowest-similarity pairs, notably populations 25 (Norðurland vestra), 1 (Vestmannaeyjar), 7 (Eyrarbakki), and 18 (Stykkishólmur), consistent with their separation in the multivariate space.

**Table 2:** Evaluation of ISSR polymorphism and genetic variation of all of tested *L. nootkatensis* populations.

Parameter/Index	Values
AEI P	32.1 35.8
%P	100.0
PIC	0.188
(range)	(0.058–0.500)
I	0.270
(range)	(0.180–0.358)
Si	0.306
(range)	(0.098–0.606)

AEI Assay Efficiency Index; P average number of polymorphic loci; %P the percentage of polymorphic loci; PIC Polymorphism Information Content; I value of Shannon Index; Si Genetic Similarity Index.

### Population structure inferred by clustering

**Fig. 1:**
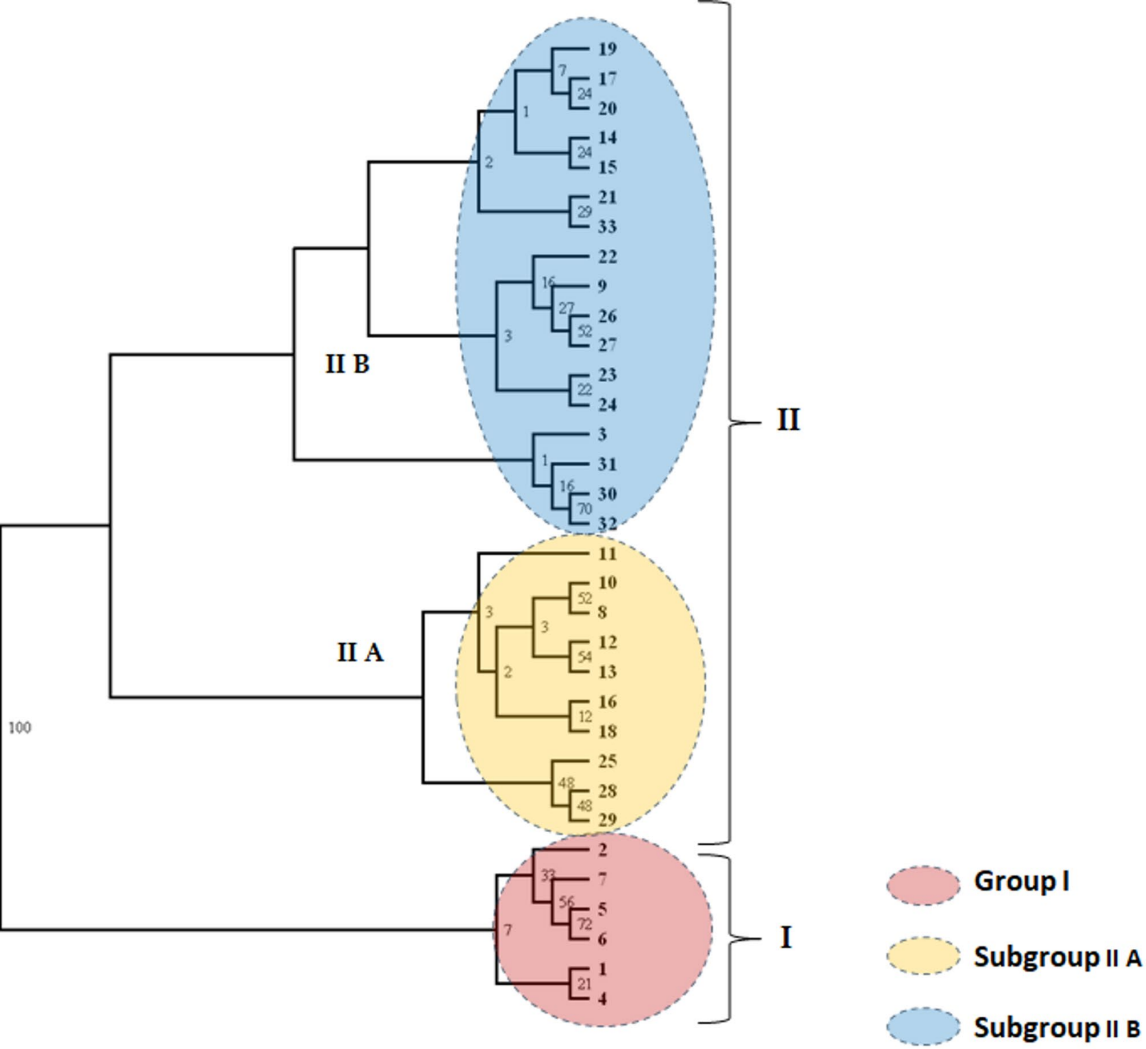
UPGMA dendrogram illustrating the genetic similarity among the 33 studied populations of *L. nootkatensis* based on ISSR markers (the horizontal axis indicates the genetic distances at which groups were joined during clustering, and population numbers correspond to those listed in Table 4). Values near the branches (500 replicates) indicate bootstrap support (BS).

The UPGMA dendrogram based on Dice similarity (Fig. 1) separated populations into two main clusters. Genetic relationships, according to the UPGMA tree clustering, were also visualised on the location map of the studied populations (Fig. 3B). Cluster I (marked in pink) contained six populations: five from the Southern Region (populations 2, 4, 5, 6, and 7) and the Vestmannaeyjar population (population 1). Cluster II comprised the remaining 27 populations and was divided into two main subgroups (II A and II B). Subgroup A (marked in yellow) included ten populations: populations 8 and 10 from the Southern Region, populations 11–13, 16, and 18 from the Western Region, and populations 25, 28, and 29 from northern Iceland. Subgroup B (marked in blue) was further structured into three aggregates. The first aggregate comprised populations 30, 31 and 32 from the Northeastern Region together with population 3 from the Southern Region. The second aggregate encompassed populations from the Westfjords and northern regions, as well as population 9 from the Southern Peninsula. The third aggregate was dominated by Western Region populations 14–20 and included population 21 from the Westfjords and population 33 from the Northeastern Region. To validate the clustering patterns, Principal Coordinates Analysis (PCoA) based on Jaccard distances was performed.

**Fig. 2:**
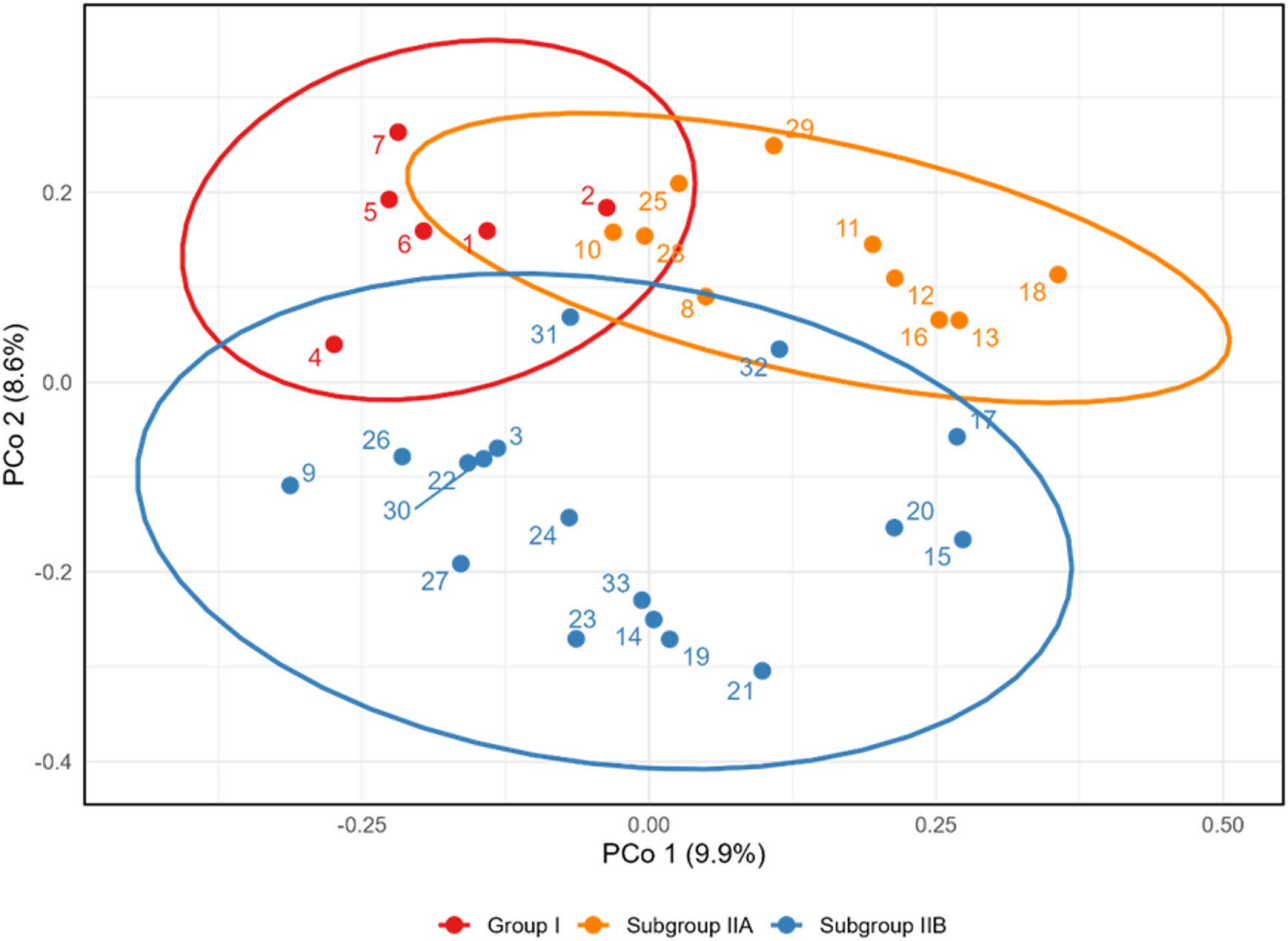
Principal Coordinates Analysis (PCoA) of 33 *Lupinus nootkatensis* populations in Iceland based on Jaccard’s genetic distances. Point colours indicate the genetic cluster membership identified by the UPGMA dendrogram: Group I (red), Subgroup IIA (orange), and Subgroup IIB (blue). Ellipses represent 95% confidence intervals around each genetic lineage. Numbers adjacent to the data points correspond to the sampled population IDs. The percentages on the axes indicate the proportion of the total genetic variance explained by the first two principal coordinates.

The Principal Coordinates Analysis (PCoA), colour-coded according to UPGMA clusters, revealed that the identified genetic lineages occupy distinct sectors of the multivariate space (Fig. 2). Although the clusters are diffuse and partially overlap, the 95% confidence ellipses confirm a statistically recognisable separation of Group I, Subgroup IIA, and Subgroup IIB. To quantify the partitioning of genetic variation and test the strength of the identified structures, Analysis of Molecular Variance (AMOVA) was conducted under two different grouping scenarios (Table 3). First, populations were grouped strictly by geographical regions (South, West, North). This categorisation was based on Iceland’s main coastal sectors, reflecting broad spatial separation. Under this geographical scenario, only 5.91% of the total molecular variance was attributed to differences among regions (Φ-statistic = 0.059, *p* < 0.001). Most of the diversity (94.09%) resided within populations.

**Table 3:** Analysis of Molecular Variance for *L. nootkatensis* populations in Iceland based on ISSR markers.

Grouping scenario	Source of variation	d.f.	Sum of squares (SS)	Variance components	% of variation	*p*-value
1. Geographical regions (South, West, North)	Among regions	2	1.076	0.02	**5.91**	< 0.001
	Within populations	30	9.644	0.321	94.09	
	Total	32	10.72	0.341	100	
2. Genetic clusters (Clusters I, IIA, IIB)	Among clusters	2	1.486	0.043	**12.32**	< 0.001
	Within populations	30	9.234	0.308	87.68	
	Total	32	10.72	0.351	100	

**Fig. 3:**
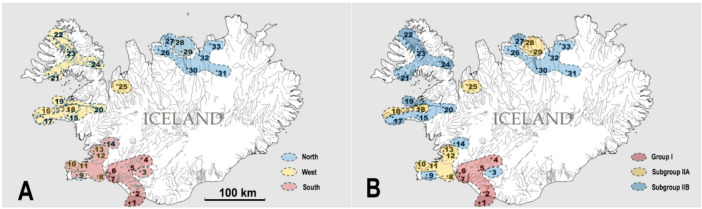
Spatial distribution of *L. nootkatensis* populations in Iceland. (**A**) Geographical grouping: populations were assigned to three broad sectors (South, West, and North) based on major coastal divisions and spatial proximity. (**B**) Genetic grouping: populations were assigned to the three main clusters identified by UPGMA. Note that while regional designations in panel A follow broad geographical sectors, the lack of strong spatial structure was further confirmed by a Mantel test, which assessed geography as a continuous variable independent of these regional categories. The map was generated by the authors using MapInfo Professional v. 11.0 software (Pitney Bowes Software, USA; https://www.precisely.com/product/mapinfo-pro) by manual vectorisation of a public domain outline map of Iceland.

In the second scenario, populations were grouped according to the genetic clusters identified in the UPGMA analysis (Group I, IIA, and IIB) (Fig. 3B). In this configuration, the proportion of variance explaining the structure more than doubled to 12.32% (Φ-statistic = 0.123, *p* < 0.001), with 87.68% of the variance remaining within populations. This significant difference suggests that the genetic structure of *L. nootkatensis* in Iceland is better explained by the identified genetic lineages – likely reflecting introduction history and dispersal pathways – than by current geographical proximity.

### Spatial genetic structure and isolation-by-distance

To address the potential arbitrariness of the categorical regional designations and further assess the strength of the geographic pattern, we performed a Mantel test comparing the Jaccard pairwise genetic distance matrix with continuous pairwise geographic distances (in km) among all 33 populations. The analysis showed a statistically significant yet very weak positive correlation (Mantel statistic *r* = 0.245, *p* = 0.0001, based on 9999 permutations). This outcome indicates that geographic distance accounts for only about 6% of the observed genetic variation (r2 ≈ 0.06). This continuous spatial analysis directly supports the AMOVA results, in which geographic regions explained just 5.9% of the total variance, confirming that natural isolation-by-distance is not the main factor driving the genetic structure of *L. nootkatensis* in Iceland.

## Discussion

A striking finding of this study is the maintenance of high genetic diversity within the Icelandic populations of *L. nootkatensis*. Introduced populations frequently experience genetic bottlenecks and reduced genetic variability compared to their native ranges. However, our ISSR data reveal substantial polymorphism (100% at the dataset level) and high gene diversity (mean Shannon’s index I = 0.270). Crucially, the AMOVA results demonstrate that the vast majority of this variation (87.7%) resides within individual populations.

This genetic pattern suggests that the Nootka lupin in Iceland has successfully mitigated the classic genetic paradox of biological invasions^32,33^. The exceptionally high standing genetic variation is a classic signature of high propagule pressure, resulting from multiple, independent introductions across various locations using material from diverse sources^34^. The systematic and massive seeding programs conducted across Iceland effectively bypassed typical founder effects, providing the species with a broad genetic base. This aligns with established invasion theory: if a species is introduced to a new region multiple times, the genetic diversity of the new combined population can be restored or even increased compared with the native range^35–37^.

Given the complexity of these historical sowing events, attempting to reconstruct the precise timeline and routes of every introduction is now futile. Instead, this study presents a possible picture of the invasion status after mass introductions have largely ceased. The current genetic structure reflects those lineages that have persisted and expanded after passing the filter of the sub-arctic climate and local soil conditions. This anthropogenic mixing likely facilitated widespread genetic admixture^38^. While our study relies on neutral ISSR markers, which preclude direct inferences regarding specific functional traits, the high genome-wide diversity generated by this admixture undoubtedly provided a robust putative basis for the species’ establishment across varied Icelandic environments^39–41^.

The spatial genetic structure provides the most compelling evidence for the invasion dynamics of *L. nootkatensis*. Both the unconstrained PCoA and categorical AMOVA demonstrated a lack of strong regional structuring, with geographical regions accounting for only 5.9% of the genetic variance. Crucially, the Mantel test revealed a significant but remarkably weak isolation-by-distance (IBD) signal, explaining merely ~ 6% of the spatial genetic variation. This methodological concordance indicates that while some localised, natural seed dispersal occurs around initial planting sites, it is vastly overshadowed by broad-scale, human-mediated admixture.

For decades, state agencies (e.g., the Soil Conservation Service of Iceland; Landgræðsla ríkisins) and forestry services actively propagated *L. nootkatensis* to combat severe soil erosion^42,43^. It is important to emphasise that these extensive translocations were driven by the urgent ecological need to stabilise the landscape at a time when modern molecular monitoring tools were unavailable. Consequently, while the primary goal of stopping erosion was achieved, these human interventions acted as a hyper-vector that completely overrode natural spatial gradients, mixing lineages from different sources and accelerating the species’ island-wide expansion.

The spatial configuration of this highly admixed population further reinforces the link between human activity and genetic structure. Notably, the highest levels of genetic complexity were observed in the Southwest region, which encompasses the Greater Reykjavík Area. Iceland is demographically unique, with approximately 64% of the total population residing in the capital^44^. While the history of this genetic diversity is complex, it is relevant that the importation of *L. nootkatensis* seeds from North America occurred primarily via Reykjavík. The high concentration of human activity in this region likely facilitated the accumulation of diverse genotypes, which were then redistributed to remote parts of the island via the radial road network.

The juxtaposition of genetically distinct lineages (e.g., Subgroup IIA and IIB) within the same regions creates ideal conditions for intraspecific admixture^45^. While such mixing in biological invasions can sometimes lead to novel genetic combinations and hybrid vigour, our use of neutral molecular markers does not allow us to directly quantify specific fitness advantages. Nevertheless, this highly interconnected population structure suggests a highly dynamic colonisation process that complicates management efforts.

Thus, while this study cannot reverse the historical introductions, it provides a critical genetic baseline to support informed decision-making. Our data suggest that the perspective of *L. nootkatensis* as merely a uniform tool for reclamation warrants re-evaluation. We now possess the evidence that human-mediated transport has transformed the population into a highly diverse metapopulation. Therefore, future management strategies would benefit from evolving beyond purely spatial containment to include measures that limit further anthropogenic mixing of these genetic lineages^46^.

Our findings provide a significant refinement of the genetic baseline established by previous research. An earlier study using SNP markers derived from the nuclear ITS2 region reported relatively low genetic differentiation among Icelandic populations^25^. That pattern is consistent with the nature of the ITS2 marker, which is evolutionarily conserved and widely used for species identification (barcoding) rather than for detecting recent intraspecific divergence. The level of genetic variation in another invasive species, *L. polyphyllus*, within and between populations on the East European Plain was examined by^26^ using several molecular markers. As in previous studies^25^, the ITS1–2 sequences were uninformative markers of intraspecific variation, whereas other markers (chloroplast rpl32–trnL) showed high levels of variation. However, the ISSR sequences of 38 individuals showed statistically significant interindividual variation both within (0.248) and between local populations (0.226) (mean, Dice coefficient). In the present study, a similar mean Dice coefficient (0.306) was obtained, thereby confirming significant variability between the studied *L. nootkatensis* populations.

In contrast to conserved loci, the ISSR markers employed in this study target hyper-variable regions of the genome (microsatellites). This higher resolution allowed us to uncover substantial cryptic diversity that was previously masked. The detection of 100% polymorphism and high Shannon’s indices confirms that while the *L. nootkatensis* invasion might appear uniform at conserved loci, it is highly dynamic at the micro-evolutionary scale. This highlights the necessity of using rapidly evolving markers to accurately assess recent biological invasions. Evidence that the lupin genome is very rich in (AG), (GA), (CA), (AC), and (GT) repeat motifs is provided by^47^, which was confirmed in the present analyses. The highest number of polymorphic ISSR-PCR amplification products was observed in the presence of dinucleotide repeat sequences (AG)_8_, anchored at the 3’ end. However, microsatellites with repeats (CA)_8_, (AC)_8_, and (CA)_7_, were found to be the most informative in detecting ISSR polymorphisms, as evidenced by higher PIC values compared to the other primers.

It seems that *L. nootkatensis* possesses a broad genetic repertoire. The species can likely withstand significant environmental stress and continue to expand. Because this high genetic diversity translates to a high capability for further spread, the lupin poses a severe and ongoing danger to vulnerable native ecosystems. Therefore, active human-mediated management is urgently required to protect other pristine environments, such as unique moss-heathlands, from being irreversibly transformed. Consequently, conservation resources might be used most effectively by shifting from a broad-scale control strategy to one of targeted containment and asset protection.

This documented extent is compatible with the expectation that extensive and repeated introductions, followed by secondary spread, have produced a spatially complex invasion footprint.

Based on our genetic data, two specific directions for future management emerge:

- Limiting Further Anthropogenic Admixture: Since human transport is a primary driver of the genetic structure, measures to minimise the movement of soil and machinery between distinct regions (e.g., from the South to the Westfjords) would be highly beneficial. This would slow the mixing of lineages and reduce the spread of highly admixed, potentially robust genotypes.
- Protecting High-Value Habitats: Given the species’ robust genetic health, efforts should likely prioritise the defence of pristine, high-value ecosystems (such as unique moss-heathlands or rare plant communities) rather than attempting to clear the species from already heavily transformed reclamation sites.

In summary, based on the high-resolution ISSR analysis of the *L. nootkatensis* population across Iceland, we conclude:

1. High Propagule Pressure and Mitigation of Genetic Bottlenecks: Contrary to expectations for an introduced species on an isolated island, the Icelandic population of Nootka lupin retains high levels of genetic diversity. This confirms that the invasion was not driven by a limited number of founders, but by massive, repeated introductions that have effectively mitigated typical genetic bottlenecks.
2. Human-Mediated Transport Overrides Natural Dispersal: Our study empirically confirms that the colonisation pattern of *L. nootkatensis* in Iceland does not follow natural dispersal mechanisms. Geographic distance explains only a marginal fraction of the genetic variance (~ 6%). Instead, human-mediated transport has acted as a hyper-vector, bypassing biological limitations and facilitating the large-scale mixing of distant gene pools.
3. High Genetic Diversity Complicates Biological Control: The co-occurrence of genetically distinct lineages within the same regions, driven by historical translocations, has created a highly admixed population. This extensive intrapopulation diversity complicates the potential use of biological control agents (e.g., specific pathogens or herbivores), as the probability of resistant genotypes being present within the standing variation is significantly maximised.
4. The Indispensable Role of Genetic Monitoring: Most importantly, this study highlights that effective management of *L. nootkatensis* cannot rely solely on ecological observations; it requires a genetic perspective. We demonstrate that cost-effective molecular tools can reveal hidden practical mechanisms, such as the scale of anthropogenic mixing and cryptic diversity, that directly impact control efficacy.

## Materials and methods

### Plant material

Sampling was conducted across Iceland to represent the main regions in which *L. nootkatensis* occurs as an introduced and invasive species. Preliminary field identification of *L. nootkatensis* was performed by Jakub Skorupski, Ph.D. based on morphological keys^48^. Since the sampling was non-destructive and focused only on leaves for DNA extraction, traditional herbarium vouchers were not collected. Instead, species identity was confirmed via DNA barcoding, as detailed in our previous study^25^. The DNA isolates, serving as molecular vouchers, were prepared by Magdalena Szenejko, Ph.D., and are deposited at the Institute of Marine and Environmental Sciences, University of Szczecin, available upon request (vouchers no. IS_2017_DNA_1-33-66).

A total of 33 sampling sites were selected to represent the geographic range of the species in the country, including southern (Suðurland), western (Vestfirðir, Vesturland, Höfuðborgarsvæði, Suðurnes), and northern (Norðurland vestra, Norðurland eystra) regions, as well as the Vestmannaeyjar archipelago (Table 4). Samples were collected exclusively from non-protected, public areas with no access restrictions. At each sampling site, leaves were collected from five spatially separated plants to reduce the probability of sampling clonally related ramets. The collection method (leaf picking) caused no soil disturbance or damage to plant populations. All procedures complied with relevant institutional, national, and international guidelines, including the IUCN Policy Statement on Research Involving Species at Risk of Extinction. Leaf material was air-dried and stored at room temperature until further processing.

### DNA isolation

For each sampling site, total genomic DNA was extracted from each plant (*n* = 5) using the Plant DNA Mini Kit (Syngen Biotech, Poland) according to the manufacturer’s protocol optimised for dried plant tissues. The quality and quantity of the extracted DNA were assessed by 1.0% agarose gel electrophoresis and spectrophotometric analysis using a NanoDrop 2000 C spectrophotometer (Thermo Fisher Scientific, USA). Next, equal amounts of DNA from the plants were then combined to generate a single composite DNA sample representing each site for the ISSR analysis. This composite approach was applied to standardise site-level comparisons while incorporating multiple plants collected at each sampling site.

### ISSR amplification

Inter-simple sequence repeat analysis was performed according to the modified protocol of^27^.

A total of 80 ISSR primers were initially screened and obtained from the Genomed S.A. (Poland) and the Oligo IBB PAN (Poland) biotechnology platforms. Based on reproducibility, banding pattern quality, and polymorphism level, eight primers were selected for PCR (Table 5). Each PCR reaction was carried out in a final volume of 20 µL, containing 2× Phire Plant Direct PCR Master Mix (Thermo Fisher Scientific, USA), Dilution Buffer, 0.8 µM of a single ISSR primer, and approximately 30 ng of template DNA. The reaction volume was adjusted with nuclease-free water.

**Table 4:** Origin and number of tested *L. nootkatensis* populations.

Population no.	Sampling site	Region of Iceland
1	Heimaey	Westman Islands (Vestmannaeyjar)
2	Landeyjahöfn	Southern Region (Suðurland)
3	Gunnarsholt	
4	Hella	
5	Geysir	
6	Óseyrartangi	
7	Eyrarbakki	
8	Strandarkirkja	
9	Grindavík	Southern Peninsula (Suðurnes)
10	Sandgerði	
11	Vogar	
12	Reykjavík	Capital Region (Höfuðborgarsvæði)
13	Kjós	
14	Botnsdalur	Western Region (Vesturland)
15	Vegamót	
16	Útnesvegur	
17	Rif	
18	Kirkjugarður Stykkishólms	
19	Stykkishólmshöfn	
20	Sauðafell Guesthouse	
21	Bjarkarholt	Westfjords (Vestfirðir)
22	Bolungarvík	
23	Hvítanes	
24	Hólmavík	
25	Laugarbakki	Northwestern Region (Norðurland vestra)
26	Stífluvatn	
27	Siglufjörður	Northeastern Region (Norðurland eystra)
28	Ólafsfjörður	
29	Hauganes	
30	Akureyri	
31	Skútustaðir	
32	Fljótsbakki	
33	Húsavík	

Amplifications were conducted in a T100™ Thermal Cycler (Bio-Rad, California, USA) using the following thermal profile: initial denaturation at 98 °C for 5 min, followed by 40 cycles of denaturation at 98 °C for 5 s, primer annealing at 45–54 °C for 5 s (primer-specific annealing temperature optimised using a gradient thermal cycler), and extension at 72 °C for 20 s, with a final extension step at 72 °C for 1 min. Amplification products were separated by electrophoresis on 2.5% agarose gels stained with ethidium bromide (5 µg mL⁻¹; Sigma-Aldrich, Germany) in TAE buffer for 7 h. Fragment sizes were estimated using the NZYDNA Ladder VII (Nzytech Genes & Enzymes, Portugal).

**Table 5:** Characteristics of the eight primers used in this study; Y = T or C, B = C, G or T, D = A, G or T.

Primer name	Microsatellite	Sequence 5’-3’	Annealing temperature (°C)
2	(CA)_6_AT	CACACACACACAAT	45
5	(AG)_8_CC	AGAGAGAGAGAGAGAGCC	52
7	(CA)_8_AC	CACACACACACACACAAC	52
M09	(AC)_8_YG	ACACACACACACACACYG	52
807	(AG)_8_T	AGAGAGAGAGAGAGAGT	52
810	(GA_)8_T	GAGAGAGAGAGAGAGAT	54
817	(CA)_8_A	CACACACACACACACAA	54
888	BDB(CA)_7_	BDBCACACACACACACA	54

### Scoring of ISSR markers

ISSR amplification profiles were visualised and documented using the Gel Doc™ XR+ system (Bio-Rad, USA) and analysed with Image Lab™ Software 4.0 (Bio-Rad). Clear, reproducible bands were scored manually as binary characters, with presence coded as 1 and absence as 0. Only consistently amplified bands across replicate reactions were included in the final data matrix.

### Data analysis

Genetic diversity parameters were calculated from the binary ISSR presence/absence matrix. The polymorphism information content (PIC) for dominant markers was estimated according to^29^, and the assay efficiency index (AEI) was calculated as described by^28^.

For dominant markers, PIC was calculated as PIC = 1 − p² − q², where *p* is the band (presence) frequency, and *q* is the no-band (absence) frequency. AEI, which reflects the average number of polymorphic products detected per primer, was also computed. For the complete dataset, the number of polymorphic loci (P), the percentage of polymorphic loci (%P), and Shannon’s information index (I) were determined. Shannon’s index was calculated as I = −∑ p_i_ log₂(p_i_), where *pi* is the frequency of the i-th allele. Pairwise genetic similarity (S_ij_) was computed using Dice’s coefficient^49^, as implemented by^50^, according to S_ij_ = 2N_ij_/(N_i_ + N_j_), where *Nij* is the number of bands shared by samples *i* and *j*, and *Ni* and *N*_j_ are the numbers of bands present in samples *i* and *j*, respectively. The pairwise similarity matrix was used to construct a dendrogram with the unweighted pair group method with arithmetic mean (UPGMA), and a bootstrap test (500 replicates) was used to assess branch support. Cluster analysis was performed in FreeTree, and dendrogram visualisation was carried out using TreeView v. 1.6.6^51,52^.

Multivariate statistical analyses were conducted in the R computing environment^53^. Principal Coordinate Analysis (PCoA) was performed based on Jaccard distances using the vegan package^54^ and visualised with ggplot2^55^. Analysis of Molecular Variance (AMOVA) was carried out to estimate variance components and the Φ-statistic significance (*p* < 0.001) based on 1000 permutations, using functions implemented in the vegan and pegas packages^56^. Finally, to explicitly test for an isolation-by-distance (IBD) pattern, a Mantel test was performed to assess the correlation between genetic and geographic distances. The geographic distance matrix (in kilometers) was calculated from the coordinates of the sampled populations using the distm function (with the distGeo algorithm) implemented in the geosphere R package^57^. The Mantel test was then executed using the mantel function from the vegan package^54^, comparing the geographic distance matrix against the Jaccard pairwise genetic distance matrix. The statistical significance of the correlation was evaluated using Pearson’s method with 9999 permutations. Geographic Information System (GIS) analyses and visualisations were performed using MapInfo v. 11.0 (Pitney Bowes Software, USA) tool set.

### Permissions statement

No specific permits were required as *L. nootkatensis* is an invasive species in Iceland and is not subject to protection. The study did not involve species at risk of extinction or CITES-listed taxa (the Convention on International Trade in Endangered Species of Wild Fauna and Flora). Consequently, CITES permitting was not applicable.

## Supplementary Information

Below is the link to the electronic supplementary material.

Supplementary Material 1

Supplementary Material 2

Supplementary Material 3

## Data Availability

The data supporting the findings of this study are available from the author upon request.

## References

[1] Wąsowicz, P., Przedpelska-Wasowicz, E. M. & Kristinsson, H. Alien vascular plants in Iceland: Diversity, spatial patterns, temporal trends, and the impact of climate change. *Flora - Morphology Distribution Funct. Ecol. Plants*. **208**, 648–673 (2013).

[2] Guðjohnsen, S. K. & Magnússon, B. Útbreiðsla og flatarmál lúpínubreiða á Íslandi 2017 (NÍ-19001). Icelandic Institute of Natural History.(accessed 6 January 2026) Preprint at (2019)

[3] Vetter, V. M. S., Tjaden, N. B., Jaeschke, A., Buhk, C., Wahl, V., Wąsowicz, P. & Jentsch, A. Invasion of a legume ecosystem engineer in a cold biome alters plant biodiversity. *Front. Plant. Sci.* **9**, 715 (2018). 10.3389/fpls.2018.00715 PMC5996276 29922310

[4] Baldursson, S. Fertilization and seed set in Nootka lupine. in *Biological Studies of Nootka lupine (Lupinus nootkatensis) in Iceland. Growth, Seed Set, Chemical Content and Effect of Cutting.* (ed. Magnusson, B.) vol. 178, pp. 38–43 (1995).

[5] Magnusson, B. NOBANIS – Invasive Alien Species Fact Sheet – Lupinus nootkatensis. *Online Database of the European Network on Invasive Alien Species.* (2010). https://www.nobanis.org/globalassets/speciesinfo/l/lupinus-nootkatensis/lupinus_nootkatensis.pdf*– NOBANIS.* 1–12.

[6] Hautala, R. & Kumala, J. How erosion and other natural forces have changed the Icelandic landscape–is Nootka lupine fixing any of these problems? *Suoseura* **74**, 119–127 (2023).

[7] CAFF & PAME. Arctic Invasive Alien Species: Strategy and Action Plan. Conservation of Arctic Flora and Fauna (CAFF) & Protection of the Arctic Marine Environment (PAME). (Accessed 6 January 2026). https://www.pame.is/images/03_Projects/MPA/ARIAS/Arctic_Invasive_Alien_Species_Strategy_and_Action_Plan_ARIAS.pdf Preprint at (2017).

[8] Benediktsson, K. Floral hazards: nootka lupin in iceland and the complex politics of invasive life. *Geogr. Ann. Ser. B*. **97**, 139–154 (2015).

[9] Svavarsdóttir, K., von Schmalensee, M., Aradóttir, Á. L., Bau, A. & Stefánsson, R. A. Áhrif sláttar og eitrunar á lúpínubreiður og gróðurfar. *Náttúrufræðingurinn* **86**, 5–18 (2016).

[10] Schierbeck, G. *Skýsla Um Nokkrar Tilraunir Til Jurtaræktunar á Islandi*. (Reykjavík, 1886).

[11] Magnússon, B. M. S. H., S. B. D. *Áhrif Alaskalúpínu á Gróðurfar.* (2003).

[12] Hrafnkelsdóttir, B. The interaction between native insect herbivores, introduced plant species and climate change in Iceland. (2020).

[13] Karlsson, T. Den gammaldags lupinen in Sunnerbo. *Svensk botanisk tidsskrift*. **75**, 265–278 (1981).

[14] Fremstad, E. & Elven, R. Perennial lupins in Fennoscandia. in *Wild and Cultivated Lupins from the Tropics to the Poles. Proceedings of the 10th International Lupin Conference, Laugarvatn, Iceland, 19 – 24 June 2002*. 178–186 (International Lupin Association, Canterbury, New Zealand, 2004).

[15] Bjarnason, H. *The 50th Anniversary of [Icelandic] Forest Legislation 1907–1957.* (1957).

[16] Arnalds, A. & Runolfsson, S. The role of Nootka lupin (Lupinus nootkatensis) for revegetation in Iceland. in *Wild and cultivated lupins from the tropics to the poles. Proceedings of the 10th International Lupin Conference, 19-24 June 2002* (ed. Santen E.v, H. G. D.) 94–96 (International Lupin Association, Canterbury, New Zealand, Laugarvatn Icelan, 2004).

[17] Halldórsson, G. S. K., Þ. E. F., R. S. Vistheimt á vegum Landgræðslu ríkisins [Ecological restoration actions of the Soil Conservation Service of Iceland]. in *Vistheimt á Íslandi [Ecological restoration in Iceland]*. (ed. Aradóttir Á. L., *H G)* 40–48 (2011). (Landbúnaðarháskóli Íslands og Landgræðsla ríkisins, Reykjavík, Iceland.

[18] Runólfsson, S. Lúpínan hefur lokið hlutverki sínu [The lupin has now ended its role]. (2018). https://www.mbl.is/frettir/innlent/2018/08/16/lupinan_hefur_lokid_hlutverki_sinu/

[19] Pálmason, F., Gudmundsson, J. & Sverrisson, H. Estimates of symbiotic nitrogen fixation in two lupin species in Iceland. in *Wild and cultivated lupins from the tropics to the poles. Proceedings of the 10th International Lupin Conference, Laugarvatn Iceland, 19-24 June 2002* 118–120 (International Lupin Association, Canterbury, New Zealand, 2004).

[20] Sigurðsson, B. D. *Fræforði Alaskalúpínu (Lupinus nootkatensis) á uppgræðslusvæðum í Heiðmörk og Öræfasveit* (Háskóli Íslands, 1993).

[21] Riege, D. A. 2004. The use of Lupinus nootkatensis in a revegetation and afforestation program in Southwest Iceland. in *Wild and cultivated lupins from the tropics to the poles. Proceedings of the 10th International Lupin Conference, Laugarvatn Iceland, 19-24 June 2002*. (ed. Santen, E. van, H. G. D.) 206–207 (International Lupin Association, Canterbury, New Zealand, 2004).

[22] Sigurdsson, B. D. & Magnusson, B. Seed ecology of the Nootka lupin (Lupinus nootkatensis) in Iceland. in *Wild and cultivated lupins from the tropics to the poles. Proceedings of the 10th International Lupin Conference, Laugarvatn Iceland, 19-24 June 2002*. (ed. Santen, E. van, H. G. D.) 195–198 (International Lupin Association, Canterbury, New Zealand, 2004).

[23] Svavarsdóttir, K. , Pétursdóttir, T. & Gísladóttir, G. Distribution dynamics of exotic Nootka lupin (Lupinus nootkatensis) on a braidedriver plain in Skaftafell National Park, Iceland. in *Wild and cultivated lupins from the tropics to the poles. Proceedings of the 10th International Lupin Conference, Laugarvatn Iceland, 19-24 June 2002*.(ed. Santen, E. van, H. G. D.) 199–202 (International Lupin Association, Canterbury, New Zealand, 2004).

[24] Björnsson, D. Hörfar lúpínan? Dæmi úr Heiðmörk. *Skógræktarritið* 12–17 (2011).

[25] Skorupski, J., Szenejko, M., Gruba-Tabaka, M., Śmietana, P. & Panicz, R. Inferring population structure and genetic diversity of the invasive alien Nootka lupin in Iceland. *Polar Res.* **40**, 4536 (2021).

[26] Galkina, M. A. et al. Invasive plant Lupinus polyphyllus demonstrates high level of molecular genetic variation within and between populations at East European Plain. *Sci. Rep.* **15**, 14960 (2025). 40301434 10.1038/s41598-025-98764-9 PMC12041230

[27] Ziętkiewicz, E., Rafalski, A. & Labuda, D. Genome fingerprinting by simple sequence repeat (SSR)-anchored polymerase chain reaction amplification. *Genomics* **20**, 176–183 (1994). 8020964 10.1006/geno.1994.1151

[28] Pejić, I. et al. Comparative analysis of genetic similarity among maize inbred lines detected by RFLPs, RAPDs, SSRs, and AFLPs. *Theor. Appl. Genet.* **97**, 1248–1255 (1998).

[29] Ghislain, M., Zhang, D., Fajardo, D., Huamán, Z. & Hijmans, R. J. Marker-assisted sampling of the cultivated Andean potato Solanum phureja collection using RAPD markers. *Genet. Resour. Crop Evol.* **46**, 547–555 (1999).

[30] Szenejko, M., Śmietana, P. & Stępień, E. Genetic diversity of Poa pratensis L. depending on geographical origin and compared with genetic markers. *PeerJ* **4**, e2489 (2016). 27703847 10.7717/peerj.2489 PMC5045881

[31] Więcław, H. et al. Morphological variability and genetic diversity in *Carex buxbaumii* and *Carex hartmaniorum* (Cyperaceae) populations. *PeerJ* **9**, e11372 (2021). 34026355 10.7717/peerj.11372 PMC8121068

[32] Allendorf, F. W. & Lundquist, L. L. Introduction population biology, evolution, and control of invasive species. *Conserv. Biol.* **17**, 24–30 (2003).

[33] Frankham, R. Genetics and extinction. *Biol. Conserv.* **126**, 131–140 (2005).

[34] Lockwood, J. L., Cassey, P. & Blackburn, T. The role of propagule pressure in explaining species invasions. *Trends Ecol. Evol.* **20**, 223–228 (2005). 16701373 10.1016/j.tree.2005.02.004

[35] Rius, M. & Darling, J. A. How important is intraspecific genetic admixture to the success of colonising populations? *Trends Ecol. Evol.* **29**, 233–242 (2014). 24636862 10.1016/j.tree.2014.02.003

[36] Dlugosch, K. M. & Parker, I. M. Founding events in species invasions: Genetic variation, adaptive evolution, and the role of multiple introductions. *Mol. Ecol.* **17**, 431–449 (2008). 17908213 10.1111/j.1365-294X.2007.03538.x

[37] Estoup, A. & Guillemaud, T. Reconstructing routes of invasion using genetic data: Why, how and so what? *Mol. Ecol.* **19**, 4113–4130 (2010). 20723048 10.1111/j.1365-294X.2010.04773.x

[38] Ellstrand, N. C. & Schierenbeck, K. A. Hybridization as a stimulus for the evolution of invasiveness in plants? *Proc. Natl. Acad. Sci.* **97**, 7043–7050 (2000). 10860969 10.1073/pnas.97.13.7043 PMC34382

[39] Baker, H. G. Characteristics and modes of origin of weeds. in *The Genetics of Colonizing Species* (ed. Baker, H. G., S. G. L.) 147–168 (Academic Press, New York, 1965).

[40] Ziska, L. H., Tomecek, M. B., Valerio, M. & Thompson, J. P. Evidence for recent evolution in an invasive species, Microstegium vimineum, Japanese stiltgrass. *Weed Res.* **55**, 260–267 (2015).

[41] Barrett, S. C. H. Foundations of invasion genetics: The Baker and Stebbins legacy. *Mol. Ecol.* **24**, 1927–1941 (2015). 25442107 10.1111/mec.13014

[42] Magnússon, B., Magnússon, S. H. & Sigurðsson, B. D. *Langtímaáhrif Alaskalúpínu á Gróður Og Jarðveg á Íslandi. Náttúrufræðistofnun Íslands, NÍ-18005* (Náttúrufræðistofnun Íslands, 2018).

[43] Aradóttir, Á. L., Petursdottir, T., Halldorsson, G., Svavarsdottir, K. & Arnalds, O. Drivers of ecological restoration: Lessons from a century of restoration in Iceland. *Ecol. Soc.* **18**, art33 (2013).

[44] Statistics Iceland. Population by municipality, sex, citizenship and quarters 2011–2025 (Table MAN10001) [Data set]. px.hagstofa.is. (2025).

[45] Verhoeven, K. J. F., Macel, M., Wolfe, L. M. & Biere, A. Population admixture, biological invasions and the balance between local adaptation and inbreeding depression. *Proc. R. Soc. B: Biol. Sci.* **278**, 2–8 (2011). 10.1098/rspb.2010.1272 PMC2992731 20685700

[46] Hulme, P. E. et al. Grasping at the routes of biological invasions: A framework for integrating pathways into policy. *J. Appl. Ecol.* **45**, 403–414 (2008).

[47] Sbabou, L., Brhada, F., Alami, I. T. & Maltouf, A. F. Genetic diversity of Moroccan Lupinus germplasm investigated using ISSR and AFLP markers. *Int. J. Agric. Biol.* **12**, 26–32 (2010).

[48] Kristinsson, H. *A Guide to the Flowering Plants and Ferns of Iceland* (Málog menning, 2010).

[49] Dice, L. R. Measures of the amount of ecologic association between species. *Ecology* **26**, 297–302 (1945).

[50] Nei, M. & Li, W. H. Mathematical model for studying genetic variation in terms of restriction endonucleases. *Proc. Natl. Acad. Sci.* **76**, 5269–5273 (1979). 291943 10.1073/pnas.76.10.5269 PMC413122

[51] Pavlíček, A., Hrdá, S. & Flegr, J. Free-Tree–freeware program for construction of phylogenetic trees on the basis of distance data and bootstrap/jackknife analysis of the tree robustness. Application in the RAPD analysis of genus Frenkelia. *Folia Biol. (Praha)*. **45**, 97–99 (1999). 10730897

[52] Hampl, V., Pavlícek, A. & Flegr, J. Construction and bootstrap analysis of DNA fingerprinting-based phylogenetic trees with the freeware program FreeTree: application to trichomonad parasites. *Int. J. Syst. Evol. Microbiol.* **51**, 731–735 (2001). 11411692 10.1099/00207713-51-3-731

[53] R Core Team. R: A language and environment for statistical computing. *R Foundation Stat. Comput. Vienna Austria* (2024).

[54] Oksanen, J. et al. vegan: An R package for community ecologists. R package version 2.6-4. Preprint at (2022).

[55] Wickham, H. *ggplot2: Elegant Graphics for Data Analysis* (Springer, 2016).

[56] Paradis, E. pegas: an R package for population genetics with an integrated–modular approach. *Bioinformatics* **26**, 419–420 (2010). 20080509 10.1093/bioinformatics/btp696

[57] Hijmans, R. J. Geosphere Spherical Trigonometry. R package version 1.5–18. Preprint at (2022).

