# Supplementary material for: Human-mediated admixture shapes high genetic diversity and the invasion dynamics of Lupinus nootkatensis in Iceland
Source: Sci Rep. 2026 May 22;16:23502. doi: 10.1038/s41598-026-54580-3 (PMC13416141; doi:10.1038/s41598-026-54580-3)
Supplement: Supplementary file 1 — Supplementary Material 1 [file 41598_2026_54580_MOESM1_ESM.pdf]

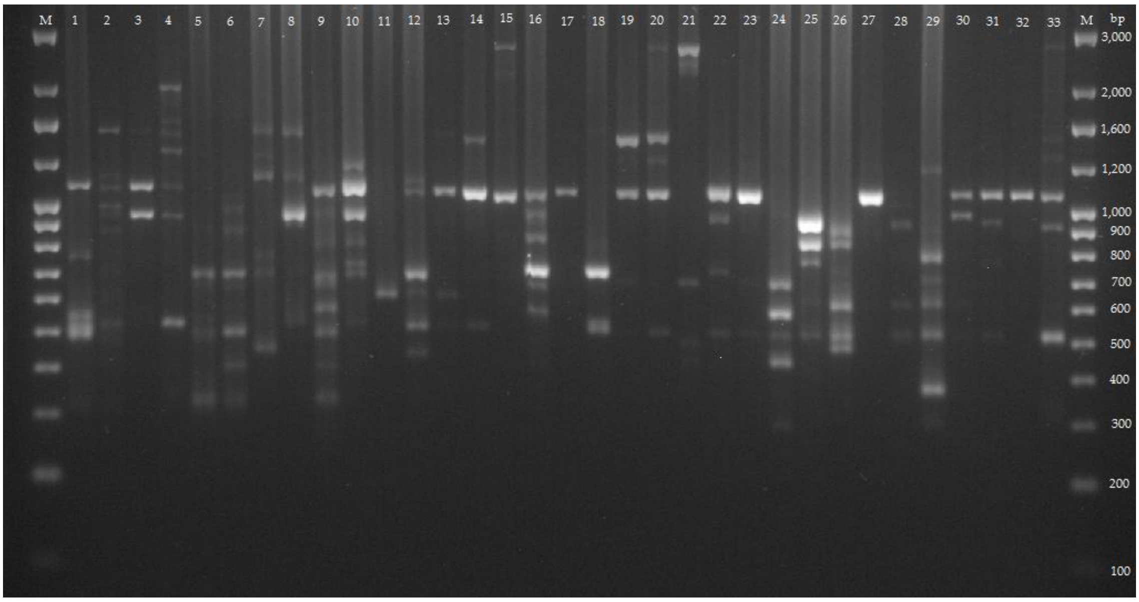

Figure S1. ISSR amplification patterns produced with primer 807 across all 33 populations of *Lupinus nootkatensis* (lanes are numbered according to the populations listed in Table 1; the ladder (M) corresponds to NZYDNA Ladder VII (Nzytech Genes & Enzymes, Portugal), serving as a molecular weight marker)
